# Unaccustomed Eccentric Contractions Impair Plasma K^+^ Regulation in the Absence of Changes in Muscle Na^+^,K^+^-ATPase Content

**DOI:** 10.1371/journal.pone.0101039

**Published:** 2014-06-24

**Authors:** Craig A. Goodman, Jason A. Bennie, Murray J. Leikis, Michael J. McKenna

**Affiliations:** 1 Institute of Sport, Exercise and Active Living (ISEAL), Muscle, Ions and Exercise Group, Victoria University, Melbourne, Victoria, Australia; 2 Department of Comparative Biosciences, School of Veterinary Medicine, University of Wisconsin-Madison, Madison, Wisconsin, United States of America; 3 Department of Nephrology, Royal Melbourne Hospital, Department of Medicine, University of Melbourne, Melbourne, Victoria, Australia; Universidade Federal do Rio de Janeiro, Brazil

## Abstract

The Na^+^,K^+^-ATPase (NKA) plays a fundamental role in the regulation of skeletal muscle membrane Na^+^ and K^+^ gradients, excitability and fatigue during repeated intense contractions. Many studies have investigated the effects of acute concentric exercise on K^+^ regulation and skeletal muscle NKA, but almost nothing is known about the effects of repeated eccentric contractions. We therefore investigated the effects of unaccustomed maximal eccentric knee extensor contractions on K^+^ regulation during exercise, peak knee extensor muscle torque, and vastus lateralis muscle NKA content and 3-O-MFPase activity. Torque measurements, muscle biopsies, and venous blood samples were taken before, during and up to 7 days following the contractions in six healthy adults. Eccentric contractions reduced peak isometric muscle torque immediately post-exercise by 26±11% and serum creatine kinase concentration peaked 24 h post-exercise at 339±90 IU/L. During eccentric contractions, plasma [K^+^] rose during Set 1 and remained elevated at ∼4.9 mM during sets 4–10; this was despite a decline in work output by Set 4, which fell by 18.9% at set 10. The rise in plasma [K^+^].work^−1^ ratio was elevated over Set 2 from Set 4– Set 10. Eccentric contractions had no effect on muscle NKA content or maximal in-vitro 3-O-MFPase activity immediately post- or up to 7 d post-exercise. The sustained elevation in plasma [K^+^] despite a decrease in work performed by the knee extensor muscles suggests an impairment in K^+^ regulation during maximal eccentric contractions, possibly due to increased plasma membrane permeability or to excitation-contraction uncoupling.

## Introduction

The Na^+^,K^+^-ATPase (NKA) plays a fundamental role in skeletal muscle contractility via the regulation of skeletal muscle membrane excitability (for review see [1], [2]). The NKA actively counteracts the action potential-induced passive influx of Na^+^ into, and efflux of K^+^ from, the muscle fiber [1], [3]. An inability to maintain the steep Na^+^ and K^+^ concentration gradients across the sarcolemmal and transverse tubular membranes during repeated intense contractions may lead to membrane depolarization and the loss of muscle force generation, referred to as fatigue [2], [4], [5]. The NKA thus acts in concert with other mechanisms that include a decline in membrane Cl^−^ conductance and intracellular acidosis, to preserve muscle membrane excitability during intense contractions [2], [5], [6].

To date, the majority of studies that have investigated the effects of acute exercise on K^+^ regulation have utilized experimental models that involve predominantly concentric muscle contractions, such as sprint or endurance cycling, and isolated knee extension exercise [7]–[11]. These studies have consistently shown a net release of K^+^ from contracting skeletal muscle, indicating an inability of the NKA to fully counteract the excitation-induced loss of K^+^ from the muscle *in-vivo* [5]. For example, during sub-maximal cycling exercise, venous and arterial plasma [K^+^] have been reported to be between 5.5–7.0 mM [12], and can exceed 8 mM during brief, high intensity treadmill exercise [13]. Micro-dialysis studies have reported muscle interstitial [K^+^] of 11–13 mM during intense concentric knee extension contractions [9], [10], [14]–[16]. In sharp contrast to these studies utilising repeated concentric contractions, almost nothing is known about K^+^ regulation during or after repeated intense eccentric muscle contractions.

During intense concentric exercise, a relationship exists between work output and plasma [K^+^], as seen during intermittent cycling exercise, where a reduction in work output was associated with a concomitant reduction in plasma [K^+^] [17], [18]. Unaccustomed eccentric, or lengthening, contractions, which are associated with increased sarcolemma and/or t-tubular membrane permeability [19], [20] have the potential to alter this relationship such that during intermittent eccentric exercise, plasma [K^+^] may increase or remain elevated despite a reduction in work output. Furthermore, acute eccentric exercise has previously been shown to reduce the abundance of important membrane transport proteins such as GLUT-4 [21], [22] and the lactate/H^+^ transporter [23]. The NKA is located in the sarcolemmal and T-tubular membranes, in association with the cytoskeleton proteins β-spectrin and ankyrin 3 [24]. Thus, there is the possibility that intense eccentric contractions may also adversely affect the abundance and/or activity of the NKA, which, in combination with increased membrane permeability, could adversely affect K^+^ regulation during exercise. To date, however, no studies have examined the effect of unaccustomed intense eccentric contractions on plasma [K^+^] regulation during exercise and on NKA content.

Therefore, this study investigated the effects of repeated sets of unaccustomed maximal eccentric contractions on K^+^ regulation during exercise, muscle force production and the content of the membrane ion transporter NKA in skeletal muscle of untrained individuals for up to 7 days after exercise. We utilised a bout of unaccustomed maximal eccentric contractions that would induce muscle damage, as indicated by a post-exercise increase in plasma creatine kinase (CK) activity, and cause a prolonged reduction in peak muscle torque production that would each recover by 7 d post-contractions. We tested two hypotheses. Firstly that there would be a progressive increase in plasma [K^+^] during repeated eccentric contractions which, together with a fall in work output, would lead to an increase in the rise in plasma [K^+^]/total work ratio (Δ[K^+^].work^−1^). Secondly, we hypothesized that repeated damaging eccentric contractions would induce a reduction in the NKA content after the eccentric contractions.

## Materials and Methods

### Ethics Statement

Six healthy, recreationally active subjects (3 M and 3 F; age, 26.3±8.1 yr; body mass, 76.3±14.9 kg; height 173.7±17 cm; Means ± SD) volunteered for the study after being informed of all risks and giving written informed consent. Ethical approval was obtained from the Victoria University Human Research Ethics Committee (application HREC 04/44; HRETH 021/04). All subjects were physically active, however, none were currently, nor had in the last 6 months, participated in resistance training.

### Overview of Exercise Trials

Subjects attended the laboratory for a total of six testing sessions. These comprised a familiarization trial; two trials to determine variability of muscle strength; the eccentric exercise trial and follow up tests at 1 d and 7 d post-eccentric exercise. All quadriceps muscle function tests were performed using an isokinetic dynamometer (Cybex Norm 770, Henley Health Care, Massachusetts, U.S.A). During the course of this study subjects were instructed to avoid involvement in any strenuous exercise activities.

### Familiarization and Variability Trials for Maximal Isometric Torque

During the first testing session, subjects were familiarized with the isokinetic dynamometer for testing maximal knee extensor isometric torque and for performing repeated maximal isokinetic contractions. During the next two testing sessions, subjects performed a test of maximal isometric peak torque of the knee extensors. These repeated tests were used to determine the subject's variability in isometric peak torque.

### Experimental Trials

At least 2 days after the last maximal isometric torque variability trial, subjects returned to the laboratory to perform repeated sets of maximal eccentric knee extensor exercise, designed to induce localised muscle fatigue and muscle damage. Quadriceps muscle function was assessed by performing maximal isometric knee extensor contractions before and immediately after the eccentric exercise bout. Venous blood samples were taken to determine exercise-induced changes in plasma [K^+^] and creatine kinase (CK), while muscle biopsies were also obtained pre- and immediately-post exercise to determine exercise-induced changes in muscle NKA content and 3-O-MFPase activity as a marker of NKA activity in resting muscle. Subjects returned to the laboratory at 24 h and at 7 d after the eccentric exercise bout for an additional maximal isometric torque test, blood sample and muscle biopsy.

### Maximal Isometric Knee Extensor Torque Test

Maximal isometric knee extensor torque was determined using an isokinetic dynamometer. Each test comprised three 5 s maximal isometric contractions at a knee joint angle of 45°, with a 30 s recovery between contractions. A visual real-time display of the isometric torque generated during each contraction was provided to each subject during the test and strong verbal encouragement was also provided to encourage the subject to exert maximal force throughout contractions. To evaluate the eccentric exercise-induced loss of muscle strength, maximal isometric peak torque of knee extensors was assessed pre- and immediately-post eccentric exercise, and at 3 h, 24 h and 7 d post-eccentric exercise.

### Maximal Eccentric Contractions

The eccentric exercise bout was also performed on the same isokinetic dynamometer and comprised 300 maximal eccentric contractions (10 sets of 30 repetitions), at a velocity of 30°/s, with a 1 min rest interval between sets. This protocol was chosen because previous studies have shown that this number of eccentric contractions induces a significant post-exercise force deficit and increase in muscle membrane permeability as indicated by an increased plasma [CK] [25]–[27]. A visual real-time display of the torque and work for each contraction was provided to each subject during the bouts and strong verbal encouragement was also provided to encourage the subject to exert maximal force throughout all eccentric contractions.

### Muscle Biopsies

A total of five muscle biopsies were taken from the middle third of the vastus lateralis muscle of each subject, at pre-, immediately-post, 3 h, 24 h and 7 d post- eccentric exercise. After injection of a local anaesthetic into the skin and fascia (1% lidocaine (Xylocaine)), a small incision was made and a muscle sample taken (∼120 mg) using a Stille biopsy needle, with suction applied to the needle via a 50 ml syringe. Each biopsy was taken by the same experienced medical practitioner from separate incisions, at a constant depth. Samples were immediately blotted on filter paper and then frozen in liquid N_2_ until assayed later for NKA content and 3-O-MFPase activity [28].

### Blood Sampling and Processing

Prior to the eccentric knee extension bout, a teflon catheter (20G, Jelco) was inserted into a dorsal hand vein for arterialised venous blood sampling, as previously described [17]. Subjects were seated on the isokinetic dynamometer 15 min before the first pre-exercise arterialized venous blood was taken. Further blood samples were taken whilst seated during the last 5 repetitions of Sets 1, 2, 4, 8 and 10 of eccentric exercise. Blood samples were analysed in triplicate measurements for plasma K^+^ concentration ([K^+^]) using an automated analyser (Ciba Corning 865, Bayer). Serum CK concentration ([CK]) was determined in arterialized venous blood sampled pre- and immediately-post eccentric exercise, and from an antecubital vein at 3 h, 24 h, and 7 d. Each sample was placed into a plain evacuated test tube, and blood allowed to coagulate for 30 min at room temperature and then centrifuged at 1,500 *g* for 10 min. The serum was removed and frozen at −20°C until analyzed in duplicate for [CK] using an Olympus GmbH AU1000 analyzer (Olympus Diagnostics, Clare). The normal reference range of [CK] using this method is 45–130 U/L.

### Muscle Na^+^, K^+^-ATPase content

Approximately 20 mg of frozen muscle was used to determine the NKA content using the vanadate–facilitated [^3^H]-ouabain method [29], [30]. Each sample was cut into small pieces of 2–4 mg wet weight. Samples were washed at 0°C for 20 min (2×10 min) in a vanadate buffer (10 mM Tris-HCl, 250 mM sucrose, 3 mM MgSO_4_ and 1 mM vanadate, pH 7.3). Muscle samples were then incubated for 2 h at 37°C in vanadate buffer with the addition of [^3^H]-ouabain (10^−6^ M, 2.0 μCi mL^−1^; Amersham Pharmacia Biotech, Castle Hill, NSW, Australia). We have previously shown that ^3^H-ouabain binding reaches saturation after 2 h of incubation in cut sections of both control and stimulated rodent skeletal muscle that are significantly larger than the pieces muscle analysed in this study [31]. Following incubation, the muscle samples were washed for 4×30 min in ice-cold vanadate buffer to remove any unbound [^3^H]-ouabain, blotted on filter paper and weighed before being soaked overnight in vials containing 500 μL 5% trichloroacetic acid and 0.1 mM ouabain. After approx. 20 h of soaking, 2.5 mL of scintillation cocktail (Opti-Fluor, Packard, Perkin Elmer, Boston, MA, USA) was added prior to liquid scintillation counting of [^3^H] activity. The content of [^3^H]-ouabain binding sites was calculated on the basis of the sample wet weight and the specific activity of the incubation medium and samples, and was expressed as pmol.g^−1^ wet wt.

### Muscle 3-*O*-MFPase activity assay

The K^+^-stimulated 3-*O*-methylfluorescein phosphatase (3-*O*-MFPase) activity assay was determined as a marker of the maximal muscle NKA activity, as previously used in human skeletal muscle in our laboratory [8], [32]. This method has recently been criticised as being an inadequate measure of NKA activity during exercise, when intracellular sodium concentration is expected to be elevated and which activates the NKA [33]. Although the method is Na^+^-insensitive, it remains valid as a marker of NKA activity in resting muscle, when intracellular Na^+^ is not raised. Further, we and others have shown that the assay can be completely inhibited by ouabain, and is therefore selective for the NKA [32], and that the 3-*O*-MFPase activity is highly correlated with the [^3^H]-ouabain binding site content [28]. Before analysis, muscle homogenates were freeze–thawed 4 times and then diluted 1/5 in cold homogenate buffer. The 3-*O*-MFPase activity was measured in an assay medium containing 5 mM MgCl_2_, 1.25 mM EDTA, 100 mM Tris and an 80 nM 3-*O*-methyl fluorescein standard at pH 7.40. A 30 *µ*l homogenate was incubated in 2.5 ml of assay medium at 37°C for 5 min before addition of 40 *µ*l of 10 mM 3-*O*-MFP to initiate the reaction. After 60 s, 10 *µ*l of 2.58 M KCl was added to stimulate K^+^-dependent phosphatase activity and the reaction was measured for a further 60 s. All assays were performed at 37°C, using continuous stirring, with data sampled at 1 Hz on a spectrofluorimeter (Aminco Bowman AB2 SLM, Thermospectronic, Madison, WI, USA). Excitation wavelength was 475 nm and emission wavelength 515 nm, with 4 nm slit widths. The 3-*O*-MFPase activity was calculated from the slope after addition of 10 *µ*m KCl minus the slope prior to KCl addition. Measurements of 3-*O*-MFPase activity data are expressed as nmol per gram wet weight.

### Statistical Analysis

Data were analysed with a one-way repeated measures ANOVA, and the Newman-Keul's Multiple Comparison post-hoc test. The agreement between the two variability trials was assessed using an intra-class correlation coefficient (ICC). Significance was accepted at p<0.05. All data was reported as mean ± SD.

## Results

### Maximal Isometric Torque

**Figure 1:**
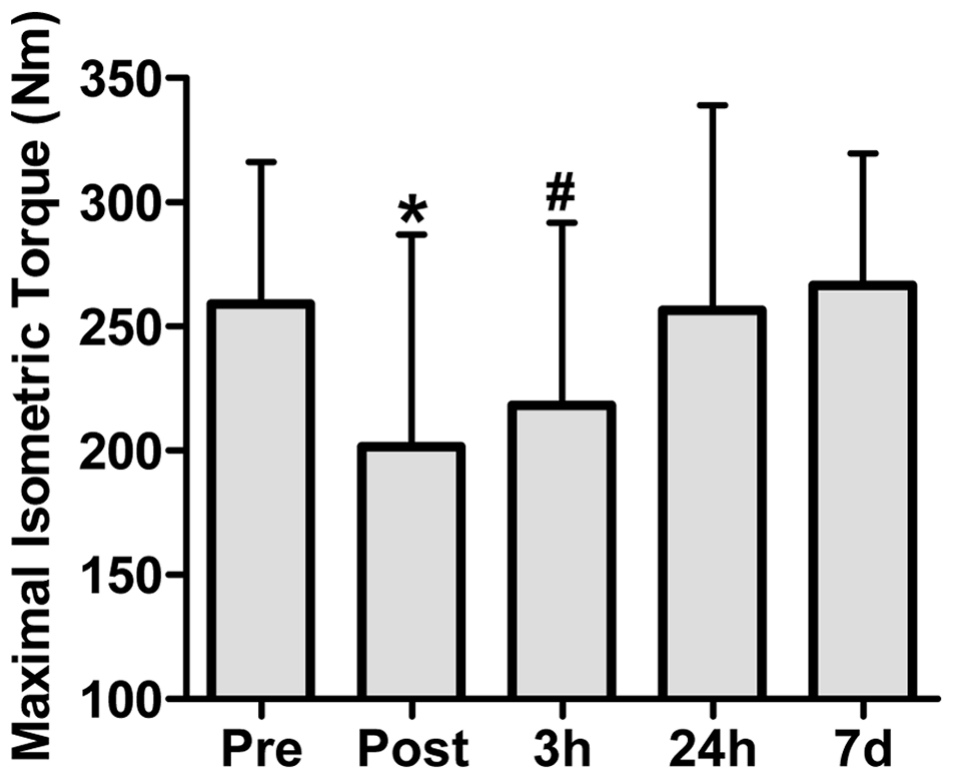
The effect of maximal eccentric knee extensor contractions on maximal knee extensor isometric torque. Measurements were taken before (Pre), immediately after (Post), and 3 h, 24 h, and 7 days following the eccentric contractions. Values are means ± SD; *n* = 6. *P<0.05– significantly different from Pre.

The maximal knee extensor isometric torque did not differ between variability trials 1 and 2 being 262±68 and 267±60 Nm, respectively. An intraclass correlation co-efficient (ICC) of 0.96 was found for peak torque for the two variability trials. The eccentric exercise bout depressed maximal isometric torque by 26±11% immediately post-exercise (P<0.05, Figure 1) and, although not statistically significant, remain depressed (19±17%) at 3 h post-exercise before returning to pre-exercise values by 24 h and at 7 d.

### Plasma [CK]

**Figure 2:**
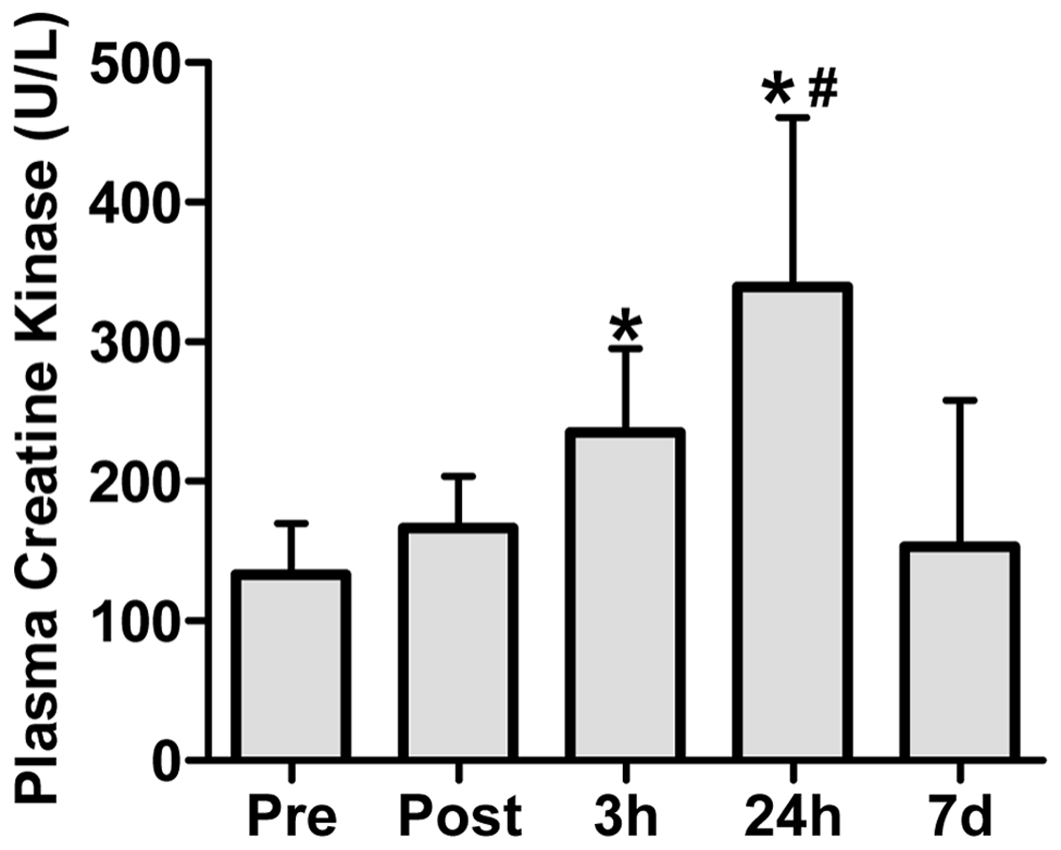
The effect of maximal eccentric knee extensor contractions on plasma creatine kinase (CK) activity. Measurements were taken before (Pre), immediately after (Post), and 3 h, 24 h, and 7 days following the eccentric contractions. Values are means ± SD; *n* = 6. *P<0.05– significantly different from Pre, Post and 7 d. # P<0.05– significantly different from all other time points.

By 3 h post-exercise, plasma [CK] was higher than Pre, Post, and 7 d post-exercise time points (P<0.05; Figure 2), and by 24 h, plasma [CK] was higher than all other time points (P<0.05). Plasma [CK] had returned to resting levels by 7 d.

### Eccentric Work and Plasma [K^+^]

**Figure 3:**
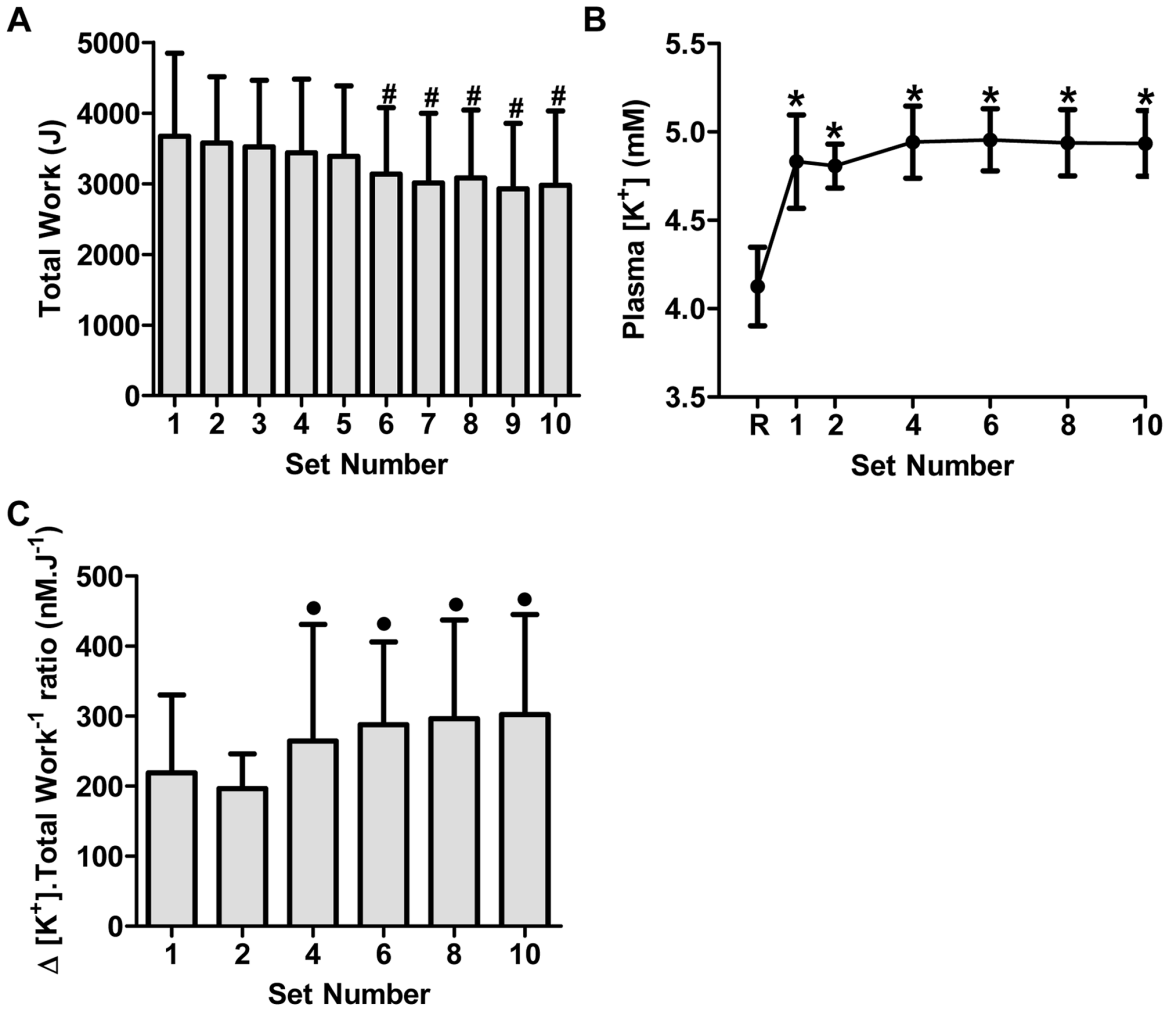
Total work performed (A), Plasma [K^+^] (B), and the rise in plasma [K^+^] relative to work performed (ΔK^+^].work^−1^ ratio) (C) during 10 sets of 30 maximal eccentric knee extensor contractions. Values are means ± SD; *n* = 6. # *P*<0.05– significantly different from Set 1. *P<0.05– significantly different from Rest. • *P*<0.05– significantly different from Set 2.

The work completed during the 10 sets of 30 repetitions of maximal eccentric exercise totalled 32,738±267 J. There were no differences in the cumulative work performed during Sets 1 to 5; however, the cumulative work performed during each of Sets 6, 7, 8, 9 and 10 was depressed by 14.6, 18.0, 16.1, 20.8 and 18.9% compared to Set 1, respectively (P<0.05, Figure 3A). Plasma [K^+^] was elevated above rest during the final contractions of Set 1 of eccentric exercise to ∼4.8 mM (P<0.05), and remained elevated during the final contractions of Sets 2, 4, 6, 8 and 10 at ∼4.9 mM (P<0.05, Figure 3B). The calculated rise in plasma [K^+^]/work ratio (Δ[K^+^].work^−1^) during eccentric exercise was elevated at Set 4 above Set 2 (P<0.05) and despite declining work output, remained elevated at this level during Sets 6, 8 and 10 (P<0.05) (Figure 3C).

### Muscle [^3^H]ouabain binding site content and maximal *in vitro* 3-*O*-MFPase activity

**Figure 4:**
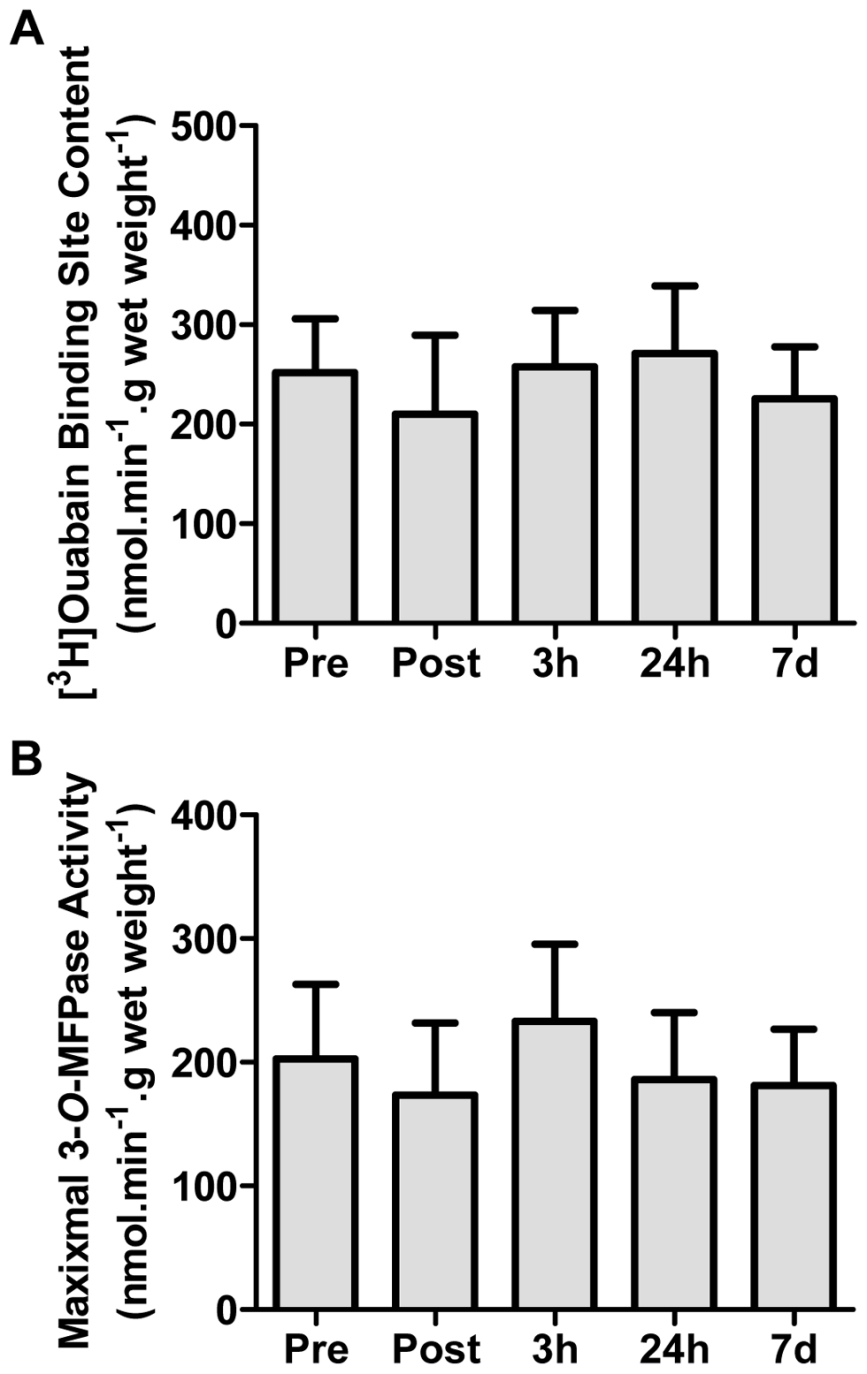
The effects of maximal eccentric knee extensor contractions on [^3^H]ouabain binding site content (a) and maximal *in vitro* K^+^-stimulated 3-*O*-methylfluorescein phosphatase (3-*O*-MFPase) activity (b). Measurements were made on muscle biopsy samples taken from the vastus lateralis muscle before (Pre), immediately after (Post), and 3 h, 24 h, and 7 days following the eccentric contractions. Values are means ± SD; *n* = 6.

There was no significant effect of eccentric exercise on [^3^H]ouabain binding site content (P = 0.314, Figure 4A) or maximal *in vitro* K^+^-stimulated 3-*O*-MFPase activity (P = 0.414, Figure 4B).

## Discussion

This is the first study to investigate the effects of unaccustomed intense eccentric contractions on K^+^ regulation during exercise and on skeletal muscle NKA content and 3-*O*-MFPase. The findings of this study supported our hypothesis that the eccentric contraction-induced increase in plasma [K^+^] would remain elevated despite a decrease in work output, resulting in an elevated Δ[K^+^]/work ratio. These data suggest that eccentric contractions, which lead to skeletal muscle damage, may impair plasma [K^+^] regulation during contractions and have an impact on intramuscular K^+^ handling. However, in contrast to our hypothesis, we found no eccentric contraction-induced decrease in muscle NKA content or the 3-*O*-MFPase as a marker of NKA activity in resting muscle.

### Evidence of muscle damage

A reduction in isometric muscle torque and an increase in plasma [CK] were used in this study as evidence for the presence of eccentric exercise-induced muscle damage [34], [35]. Whilst maximal isometric torque was depressed by 26% immediately post-exercise, this reduction in the force generating capacity post-eccentric exercise could include an element of muscle damage combined with muscle fatigue. However, the tendency for torque to remain depressed at 3 h post-exercise (19%), and the expected elevation in plasma [CK], confirms that our protocol did induce skeletal muscle damage and suggests that the integrity of the sarcolemma had been altered. The doubling of plasma [CK] at 24 h post-eccentric exercise (339±90 IU/L) in this study is similar to a previous study that also performed 300 eccentric contractions (327±156 IU/L at 24 h post-exercise) and which also demonstrated a loss of cytoskeletal protein desmin and the disruption of normal sarcomeric structure [27]. Thus, these measures confirmed that our exercise protocol was sufficient to induce a moderate level of muscle damage.

### Impaired plasma [K^+^] regulation during eccentric contractions

During concentric exercise, such as intense intermittent cycling exercise, a relationship exists between work output and plasma [K^+^] where a reduction in work output is associated with a concomitant reduction in plasma [K^+^] [17], [18]. In contrast, in the present study, plasma [K^+^] was increased above rest by the first set of eccentric contractions and, despite work output decreasing from set 6 to 10, plasma [K^+^] remained elevated for the duration of the exercise bout. As a result, the plasma [K^+^]/work ratio also remained elevated. This dissociation between plasma [K^+^] and work output suggests a possible disturbance in K^+^ regulation during the eccentric contractions, which may indicate an increased leakage of K^+^ from the muscle and/or decreased clearance of K^+^ from the plasma. This disturbance in K^+^ regulation could have contributed to a depolarizing effect on the sarcolemmal and T-tubular membranes and thus may have contributed to a fatiguing effect which, along with muscle damage, are likely responsible for the reduction in total work. Possible reasons for this disturbance in K^+^ regulation include the eccentric contraction-induced damage to the active muscle, and thus presumably also the sarcolemmal and T-tubular membranes, leading to an excess K^+^ release from the damaged muscle (and increased Na^+^ influx), or that there may have been a decrease in NKA content or activity. However, there are likely to be multiple factors contributing to muscle fatigue during eccentric contractions including an impairment in SR Ca^2+^ release [36]. In regard to the NKA content, however, we found no significant depressive effect of acute eccentric contractions immediately after exercise on NKA content as measured by [^3^H]ouabain site content. We also found no effect on the maximal NKA activity as measured by the *in vitro* 3-*O*-MFPase activity assay. Although this assay has recently been criticised as an invalid marker of NKA activity during exercise due its Na+-insensitivity, this method would appear valid for activity measures in resting muscle, as it is inhibited by ouabain and strongly correlated with [^3^H]ouabain binding measures [8], [28]. Another possible explanation for the elevated plasma [K^+^], despite a fall in work output, is eccentric contraction-induced excitation-contraction (E–C) uncoupling. In this case, eccentric contractions may have disrupted the normal communication between the dihydropyridine receptor/voltage sensor located in the T-tubular membranes and the ryanodine receptor/calcium release channels located in the sarcoplasmic reticulum membrane [37], [38]. Such a scenario would result in a fall in muscle force output due to reduced calcium release despite normal action potential propagation and associated K^+^ release [39]. Indeed, E–C coupling in mouse muscles has previously been shown to be impaired immediately post-eccentric contractions in the absence of significant changes in action potential conduction [38]–[40]. More studies are required to further explore the extent of this phenomenon in human subjects and the underlying intracellular mechanisms.

The peak plasma [K^+^] of 4.96 mmol.l^−1^ observed at the end of the 6^th^ set of eccentric contractions was similar to values previously reported during concentric contractions of knee extensor muscles on the same isokinetic dynamometer in our laboratory [8], [30]. For example, Fraser *et al*., [8] found a peak plasma [K^+^] of 4.69 mmol.l^−1^ after 50 repetitions of maximal concentric knee extension, while Petersen *et al*., [30] found a peak plasma [K^+^] of 4.89 mmol.l^−1^ after ∼ 3 min of fatiguing submaximal knee extensor contractions. The similarity in peak plasma [K^+^] between this study and Fraser *et al*., [8] and Petersen *et al*., [30], is somewhat surprising, as a unique characteristic of eccentric contractions is that they are considered not to be as metabolically demanding as purely concentric contractions [41], [42]. This decrease in metabolic demand during eccentric contractions compared to concentric is ascribed to mechanical detachment of cross-bridges during eccentric (lengthening) contractions while, in contrast, concentric contractions are dependant upon ATP- dependant cross-bridge detachment to develop force [43]. Thus, given the lower ATP utilization during eccentric exercise, it is reasonable to assume that the ionic disturbances associated with eccentric muscle contractions would also be less than during concentric contractions. Another possible reason for similar peak plasma [K^+^] during concentric and eccentric contractions may involve the enzyme AMP-activated protein kinase (AMPK). Indeed, activated AMPK has been shown to increase muscle NKA activity and lower plasma [K^+^] [44]–[46]. Therefore, the lower metabolic demand during fatiguing eccentric contractions may lead to a lower level of activation of AMPK which, in turn, may lead to a plasma [K^+^] that is similar to that found with fatiguing concentric contractions during which AMPK is substantially activated [47].

### NKA content after eccentric exercise

Previous studies have shown that an acute bout of eccentric exercise reduces the expression of the membrane-bound proteins, GLUT-4 [21], [22] and the lactate/H^+^ transporter [23]. Moreover, eccentric contractions induce the loss or disruption of cytoskeletal proteins such as dystrophin [20] and desmin [27]. Dystrophin and desmin are components of structures known as costameres which play a key role in the proper alignment of the sarcolemma and sarcomeres, and thus in lateral force transmission [48]. Importantly, the NKA is also a component of costameres and is associated with the cytoskeleton proteins, β-spectrin and ankyrin 3 [24], [48]. Thus, we hypothesized that unaccustomed eccentric exercise would lead to a decrease in NKA protein abundance as indicated by the number of [^3^H]ouabain binding sites. However, we found no significant change in [^3^H]ouabain binding sites immediately after exercise nor at 3 and 24 h post-exercise when our marker of muscle damage, plasma [CK], was elevated. Likewise, there was no sign of a compensatory upregulation of [^3^H]ouabain binding sites 7 d after eccentric exercise. These data were also supported by our finding of no significant change in NKA activity, as indicated by the maximal *in vitro* K^+^-stimulated 3-*O*-MFPase activity which, in resting muscle, has been shown to be proportional to the number of [^3^H]ouabain binding sites [8], [28]. These findings suggest that NKA protein expression may be regulated differently to other cytoskeletal/costameric proteins in response to eccentric contractions and/or that a greater level of muscle damage is required to induce a significant change in NKA expression and function. Further work is warranted to examine if a threshold of muscle damage exists, beyond which, NKA expression is changed and whether this would further alter K^+^ regulation during exercise.

One potential limitation of this study is the inclusion of both male and female subjects with a moderate range of ages. For example, sex and age related variability in NKA content or activity could potentially mask changes to these parameters in response to eccentric exercise. To date, however, there is no evidence that resting NKA content (^3^H-ouabain binding) or activity (3-*O*-MFPase activity) is different between males and females within the age range and physical activity level of the subjects in this study [see [49]–[51]). Furthermore, to the best of our knowledge there is currently no evidence that estrogens play a role in the regulation of NKA content and/or activity in skeletal muscle. Thus, based on current evidence, it is unlikely that the inclusion of both male and female subjects would have impacted NKA content or activity values found in the current study. Nevertheless, future studies should specifically investigate the existence of potential sex-based differences in potassium regulation and NKA content and activity in response to exercise, including eccentric exercise.

## Conclusion

In conclusion, eccentric contractions that led to moderate levels of muscle damage induced a dissociation between plasma [K^+^] and work output, leading to an elevated ratio of the rise in plasma [K^+^] relative to work during eccentric contractions. This most likely indicates an impairment in K^+^ regulation; however, we found no evidence of a significant change in muscle NKA content after unaccustomed eccentric exercise of the leg extensor muscles. These results may indicate the presence of excitation-contraction uncoupling during eccentric exercise. These findings highlight the need to conduct further research into K^+^ dynamics and the regulation of NKA abundance and function during eccentric exercise.
